# A deep look into radiomics

**DOI:** 10.1007/s11547-021-01389-x

**Published:** 2021-07-02

**Authors:** Camilla Scapicchio, Michela Gabelloni, Andrea Barucci, Dania Cioni, Luca Saba, Emanuele Neri

**Affiliations:** 1 Academic Radiology, Department of Translational Research, University of Pisa, Via Roma 67, 56126 Pisa, Italy; 2 CNR-IFAC Institute of Applied Physics “N. Carrara”, 50019 Sesto Fiorentino, Italy; 3 Academic Radiology, Department of Surgical, Medical, Molecular Pathology and Emergency Medicine, University of Pisa, Via Roma 67, 56126 Pisa, Italy; 4 Department of Radiology, Azienda Ospedaliero Universitaria (A.O.U.), Monserrato (Cagliari),Cagliari, Italy; 5 Italian Society of Medical and Interventional Radiology, SIRM Foundation, Via della Signora 2, 20122 Milano, Italy

**Keywords:** Radiomics, Medical imaging, Features, Imaging biomarkers, Personalized medicine

## Abstract

Radiomics is a process that allows the extraction and analysis of quantitative data from medical images. It is an evolving field of research with many potential applications in medical imaging. The purpose of this review is to offer a deep look into radiomics, from the basis, deeply discussed from a technical point of view, through the main applications, to the challenges that have to be addressed to translate this process in clinical practice. A detailed description of the main techniques used in the various steps of radiomics workflow, which includes image acquisition, reconstruction, pre-processing, segmentation, features extraction and analysis, is here proposed, as well as an overview of the main promising results achieved in various applications, focusing on the limitations and possible solutions for clinical implementation. Only an in-depth and comprehensive description of current methods and applications can suggest the potential power of radiomics in fostering precision medicine and thus the care of patients, especially in cancer detection, diagnosis, prognosis and treatment evaluation.

## Introduction

Diagnostic imaging is going through an epoch-making moment of profound transformation, to which radiologists must adapt. This is a transformation from a discipline based on the visual interpretation of the images toward a new type of radiology, which must integrate the quantitative data (biomarkers) coming from the images with the interpretative modality. In fact, since they are formed by the interaction of radiation or ultrasounds with tissues or organs, medical images are not simple images, but they reflect various physical properties of the body. Medical images can be converted into meaningful and mineable data through a quantification process. The extracted quantitative features can be analyzed to reflect the underlying pathophysiology. However, quantitative data are not easily interpretable by the human mind, they can only be extracted from a computer and analyzed through complex algorithms.

Recent advancements in the imaging domain have led to the development of processes of high-throughput extraction of quantitative features that convert images into mineable data. This process of extraction and analysis of the data, used for decision support, is named Radiomics [1, 2]. It is a promising and ongoing field of medical research that also applies state-of-the-art machine learning techniques to extract quantitative imaging features from several imaging modalities [3, 4]. By exploiting the increase in dataset size in the field of medical imaging, the extraction of quantitative features in Radiomics can be aimed to detect abnormalities in diagnostic images (e.g., lesions), as well as to follow-up pathological conditions (e.g., measuring the grow rate of lesions) or to assess treatment efficacy, with the longitudinal use of radiomics in treatment monitoring and the possibility of correcting the treatment in active surveillance. Furthermore, the extraction and the study of a huge amount of quantitative image features from radiological images could be used to predict or decode concealed genetic and molecular traits for decision support.

Despite promising results in research, such applications of radiomics still necessitate a deep exploration, refinement, standardization and validation to achieve routine clinical adoption, but they may be of great help in the clinical management of specific diseases in the near future.

Obviously, benchmarks for data extraction, analysis and presentation should be established to have reusable and repeatable results. The goal of this review is to introduce and explain the basis of radiomics and to encourage the scientific community in establishing benchmarks. The processes involved in radiomics and the reasons why it is of unique importance, as well as its challenges and their potential solutions are described here. A literature review has been performed, focusing on the latest achievements, to identify the most relevant methods used in the various studies. In the end, some of the more recent research findings and applications of importance will be mentioned, as well as a vision for radiomics of the future.

## Process

**Fig. 1:**
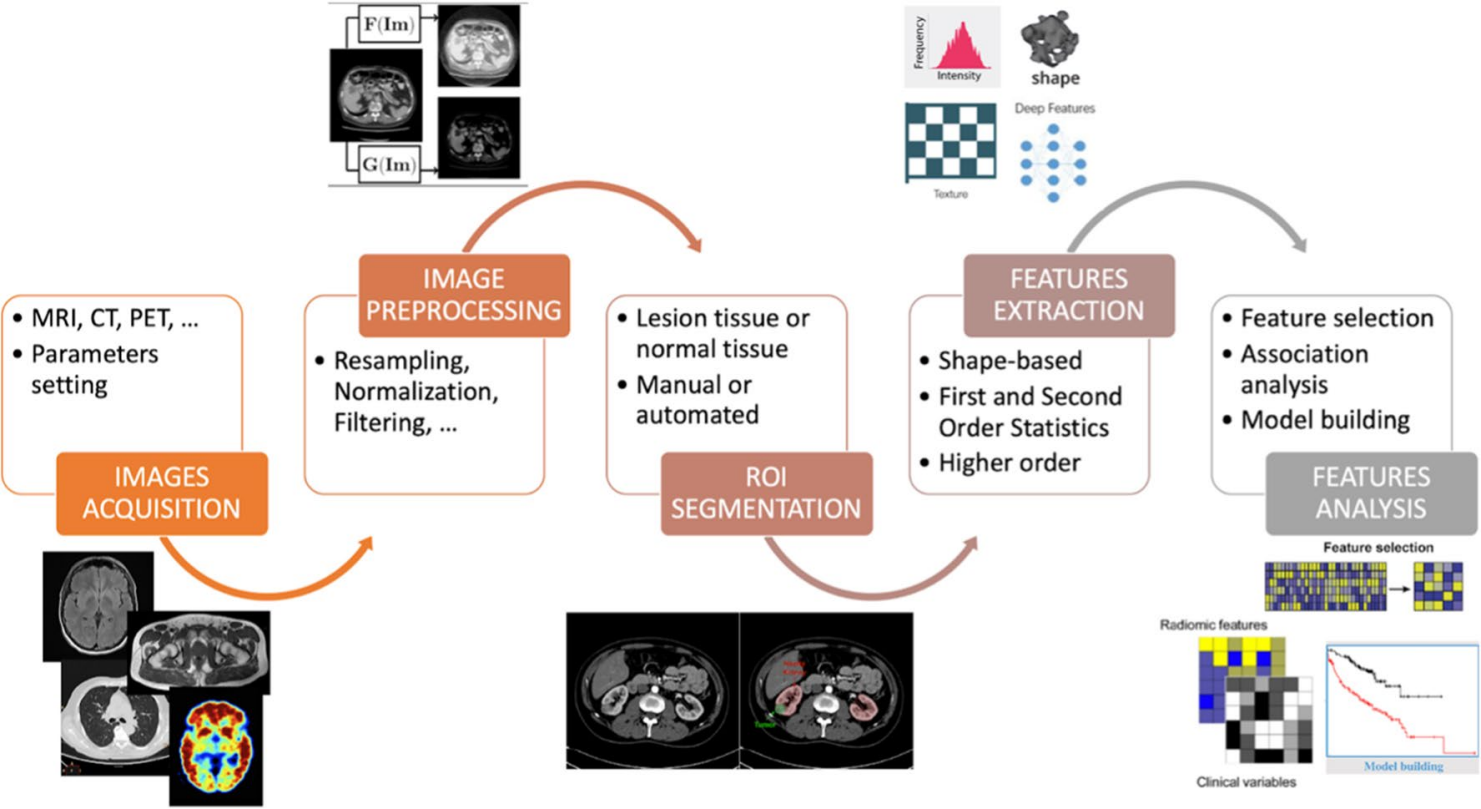
Radiomics workflow: The subsequent steps required in the radiomics process to extract radiomics features in clinical settings

The overall process of radiomics analysis requires a series of successive steps. The workflow is shown in Fig. 1. Biomedical images acquisition is the first step [1], during which several parameters have to be set, depending on the imaging modality, and therefore on the aim it is used for (diagnostic and/or treatment planning), and the tissue it has to identify. The second step involves the image pre-processing to prepare images for the following steps. Once the acquired images are pre-processed, the next step is the segmentation of the region of interest, which can be either a lesion or a normal tissue, depending on the application. The segmentation process can be accomplished manually by radiological experts or automatically by a segmentation software. The fourth step involves the extraction of radiomics features from the region of interest. A large number of features based on statistical, filtering and morphological analysis are produced, and they create a high-dimensional feature space. Then, a study on the correlation among the various features and a first analysis to identify the ‘highly’ informative features is applied, and they are selected based on user-defined criteria. The final step of a radiomics study is the use of machine learning to improve the workflow by automatically extracting and selecting the appropriate features. Machine learning algorithms are also used to build a predictive model. The model is trained on the analyzed features to learn a decision function that is used to make a prediction on previously unseen examples. The classification task of these models is defined by the user and, for instance, it can be the group characterization, the distinction of malignant tumor from benign tumors, the prediction of disease course and survival, as well as the assessment of response to therapy. All these mentioned phases of the radiomics process will be described in detail in the following paragraphs.

### Image acquisition

The biomedical images are the result of a two-dimensional and/or volumetric acquisition process, carried out with multiple modalities. Since radiomics depends on the source data, therefore the modality (x-ray, ultrasound, computed tomography, magnetic resonance, nuclear medicine), there is an intrinsic variability of the data that will be extracted from the images of the different modalities. Furthermore, within the same modality, there is a variability of acquisition according to the protocols used and the equipment [5–7].

Thus, in the numerical analysis of images, conducted to extract meaningful data, some changes could not reflect the underlying biologic effects, but they could be due to such variations in acquisitions and image reconstruction parameters. This can lead to unreliable outcomes.

Multiple initiatives have been proposed to define acquisition and reconstruction standards and thus to advance quantitative imaging in ensuring reliability. For example, the Radiological Society of North America and the National Institute for Biomedical Imaging and Bioengineering have sponsored the Quantitative Imaging Biomarkers Alliance (QIBA), the European Society of Radiology developed the so-called European Imaging Biomarkers Alliance subcommittee (EIBALL) [8, 9].

Such initiatives aim to develop a general consensus on the measurement accuracy of a quantitative imaging biomarker, and the procedures required to achieve the best level of accuracy. After acquisition, the images are progressively collected to constitute a large database and undergo a first step of pre-processing, in order to ensure uniformity and consistency.

### Image reconstruction and pre-processing

A medical image is the result of different processes, each one contributing in different and mixed ways to the final result. Understanding that what we are analyzing is not the truth, but just a representation of the real object, can be of paramount importance to build knowledge around radiomics. Clinical images are usually mathematically reconstructed from raw data acquired by the physical detectors. These raw data are a physical representation of the object under study, as observed by the interrogation system, filtered by the properties of the detectors, and by all the devices constituting the electronic acquisition and transmission chain. The acquisition of raw data is considered good if the information coming from the object under study is preserved as much as possible, despite the influence of the different parts concurring with the process [10].

The raw data must be processed to reconstruct the image as seen by the radiologist. This process is performed by using mathematical algorithms (kernels), which introduce peculiarities related to their exact formulation and implementation. Different reconstruction algorithms will introduce diversity in the radiomics analysis [11]. This step again is a sort of filtering that will affect the displayed image. Among other factors, the algorithm influences spatial resolution and the shapes inside the image.

The majority of the parameters for image reconstruction can be tweaked by the user, but some remain under a very limited control. All of these considerations highlight how the image reconstruction process must be clear if a quantitative image analysis is the goal of the study. In fact, radiomics shows an intrinsic high dependency on image parameters, such as the size of the pixel or voxels or the number and the range of the gray levels [5, 12]. For this reason, several pre-processing techniques have been proposed in order to minimize the influence of acquisition/reconstruction protocols and harmonize the images; such techniques become of paramount importance when dealing with multicentre studies [13, 14]. Some pre-processing techniques examples are shown in Table 1.

**Table 1:** Pre-processing algorithms and filters commonly used before image segmentation

Pre-processing technique	Effect on image
Resampling	Changing the number of pixels in the image using interpolation (linear, polynomial, spline, etc.)
Normalization or intensity standardization	Changing the range of pixel intensity values, in order to remove bias, scaling factors and outliers from the image
Quantization of gray levels	Reduction of gray levels used to represent the image
Motion correction	Reduction of motion confounds
Filtering to remove noise and/or improve image characteristics	Laplacian: bringing out area of rapid intensity change and usually used for edge detection Gaussian: smoothing the image and reducing noise Edge filters: resulting in edge enhancement by calculating an approximation of the derivatives in horizontal and vertical directions Laws’ filters: emphasizing image textures of edge, spot, ripple, wave, undulation and oscillation Wavelet filtering or transform methods: decomposing the original image and offering some advantages, such as variation of the spatial resolution (to represent textures at the most appropriate scale), enhancement of the texture appearance and a very wide range of choices for the wavelet function that can be adjusted for specific applications
Inhomogeneity correction	performed on MR images, where the residual effect of the variation of intensity, mainly caused by static magnetic field inhomogeneity and imperfections of the radiofrequency coils, is not eliminated by the previous normalization

### Segmentation

Segmentation is an essential step of the radiomics workflow, as highly distinctive features will be obtained from the segmented region of interest, that can be traced in a 2D image (i.e., x-ray) or in a volume (i.e., a CT volumetric acquisition); the accuracy of the segmentation will determine the radiomics features that will be extracted; segmentation differences between algorithms and operators can therefore generate an error in the creation of a radiomics map for the same area of interest [15]. Tumors may have indistinct borders, and there are still debates on how to define a reproducible ground-truth. The segmentation of a region of interest can be manual, semi-automatic or fully automatic. Manual segmentation of the tumor volume is a normal clinical procedure in the planning process before patients receive radiotherapy. It is easy, but as a drawback it is highly subjective and time-consuming. Several software and segmentation algorithms are available to perform semi-automated and fully automated segmentation on radiological images. An example is 3D-Slicer, an open-source segmentation software widely adopted in the medical research field [16]. However, since these automated tools are based on an unreal ground-truth, there is an emerging consensus that the best reliable segmentation is achievable with computer-aided edge detection followed by manual curation.

In a computer-aided detection system, the segmentation techniques commonly used are active contour, level-set, region-based and graph-based methods [17, 18]. Each algorithm can outline the region of interest for segmentation by using a different criterion. The active contour model and the level-set model are based on the prior knowledge of size, position and structure of the ROI, the region-based method relies on the principle of homogeneity and the difference between gray levels, the graph-based method exploits the variability of the pixels in the neighborhood [19]. Recently, various deep learning-based approaches, such as the Convolutional Neural Networks (CNN), have been used for medical image segmentation and demonstrated promising results [20].

### Features extraction

Feature extraction is the next step after the region of interest is segmented. It is the selection of useful information to assist in the characterization of normal and abnormal radiological images. This step is the heart of radiomics. It is worth remarking as radiomics must be considered a data-driven approach, meaning that there is no a priori hypothesis made about the clinical relevance of the features, which are computed automatically by image analysis algorithms. The purpose is to discover previously unseen image patterns using these agnostic or non-semantic features, performing classification or prediction based on the most discriminative ones, developing the so-called radiomics signature.

The features are mathematically extracted by using first-order, second-order or higher-order statistical methods, and can be generally classified in shape-based, first, second and higher-order statistics. There is no general consensus about the definition, the name, the evaluation algorithm and the belonging class, giving rise to problems when comparing different radiomics studies. In this work, we will describe features in compliance with the definitions described by the Imaging Biomarker Standardization Initiative [21].

The shape-based features are descriptors of the 2D or 3D size and shape of the region of interest and are independent from the gray level intensity distribution in the region of interest. They give a quantitative description of the geometrical characteristics of the region of interest [22]. Examples of shape-based features are shown in Table 2.

**Table 2:** Examples of 2D and 3D shape-based features with their description [23]

2D	Measure
Mesh Surface	The sum of all areas defined for each triangle in the mesh
Pixel Surface	The surface area of a single pixel multiplicated by the number of pixels in the region of interest
Perimeter	The sum of all perimeters of each line in the mesh circumference
Perimeter to Surface ratio	The ratio of the Perimeter to the Mesh Surface
Sphericity	The ratio of the perimeter of the tumor region to the perimeter of a circle with the same surface area as the tumor region and therefore a measure of the roundness of The shape of the tumor region relative to a circle
Spherical Disproportion	The ratio of the perimeter of the tumor region to the perimeter of a circle with the same surface area as the tumor region, and by definition, the inverse of Sphericity
Maximum 2D diameter	The largest pairwise Euclidean distance between tumor surface mesh vertices
Major and Minor Axis Length	The largest and the second-largest axis length of the region of interest-enclosing ellipsoid and is calculated using the largest principal component
	The inverse ratio of the major and minor principal axis lengths that could be viewed as the extent to the section is circle-like (not elongated) than it is 1 dimensional line (maximally elongated)
3D	Measure
Compactness 1 and 2	A measure of how compact the shape of the tumor is relative to a sphere (most compact)
Spherical disproportion	The ratio of the surface area of the tumor region to the surface area of a sphere with the same volume as the tumor region, and by definition, the inverse of Sphericity
Sphericity	A measure of the roundness of the shape of the tumor region relative to a sphere
Mesh Volume	the sum of all volumes defined for each face in the triangle mesh of the region of interest
Voxel Volume	The volume of a single voxel multiplicated by the number of voxels in the region of interest
Surface Area	the sum of all areas of each triangle in the mesh
Surface Area to Volume Ratio	The ratio of the Surface Area to the Mesh Volume
Maximum 3D diameter	The largest pairwise Euclidean distance between tumor surface mesh vertices
Major, Minor and Least Axis Length	Respectively, the largest, the second-largest and the smallest axis length of the region of interest-enclosing ellipsoid, calculated using the largest principal component
Elongation	The inverse ratio of the major and minor principal axis lengths that could be viewed as the extent to which a volume is longer than it is wide, i.e., is eccentric
Flatness	The inverse ratio of the major and least axis lengths that could be viewed as the extent to which a volume is flat relative to its length

First-order statistics features consider the distribution of values of individual voxels disregarding the spatial relationships [24, 25]. A normalized first-order histogram (H) can be computed from the image as follows:

1 $$H\left( i \right){\text{ }} = {\text{ }}\;{\text{No}}.{\text{ }}\;{\text{of }}\;{\text{pixels }}\;{\text{with }}\;{\text{gray}}\; - \;{\text{levels }}\;{\text{in}}\;{\text{ }}\{ I \in Bi\} {\text{ }}/\Sigma \;{\text{No}}.{\text{ }}\;{\text{pixels }}\;{\text{in }}\;{\text{the }}\;{\text{image}}$$

Being I the voxel intensity and B the equally spaced bins. From this histogram, first-order features are computed using specific equations, reducing a region of interest to a single value representation (Table 3).

**Table 3:** Examples of first-order statistics features with their description [23]

Type of feature	Measure
Energy	A measure of the magnitude of voxel values in an image
Total Energy	The value of Energy feature scaled by the volume of the voxel in cubic mm
Entropy	a measure of the inherent randomness in the gray level intensities of the image
Minimum	The lowest intensity present
Maximum	The maximum gray level intensity within the region of interest
10th and 90th percentile	The 10th and 90th percentile of the gray level intensity within the region of interest
Mean	The average gray level intensity within the region of interest
Median	The median gray level intensity within the region of interest
Range	The range of gray values in the region of interest
Interquartile Range	The range between the 25th and 75th percentile of the image array
Mean Absolute Deviation (MAD)	The mean distance of all intensity values from the Mean Value of the image array
Robust Mean Absolute Deviation (rMAD)	The mean distance of all intensity values from the Mean Value calculated on the subset of image array with gray levels in between, or equal to the 10th and 90th percentile
Root Mean Squared (RMS)	The square-root of the mean of all the squared intensity values
Standard Deviation	The amount of variation or dispersion from the Mean Value
Skewness	The asymmetry of the distribution of values about the Mean value
Kurtosis	a measure of the ‘peakedness’ of the distribution of values in the image region of interest
Variance	The mean of the squared distances of each intensity value from the Mean value and a measure of the spread of the distribution about the mean
Uniformity	The sum of the squares of each intensity value and a measure of the homogeneity of the image array

The first-order statistics values depend on the number of bins, which has to be selected not too small or too large so as the histogram may correctly represent the underlying distribution within the region of interest. It is difficult to directly compare results between studies using a different number of bins within the histograms. Optimal binning is thus a major challenge, and it depends on the pre-processing step of image quantization.

Second-order features, first introduced by Haralick [26], are based on the joint probability distribution of pairs of voxels, describing the spatial arrangement of patterns, sometimes imperceptible to the human eye. The analysis is usually performed in a double step. First a specific matrix allocating the information on the spatial distribution of pixel values is defined. Then some metrics on this matrix are evaluated.

**Fig. 2:**
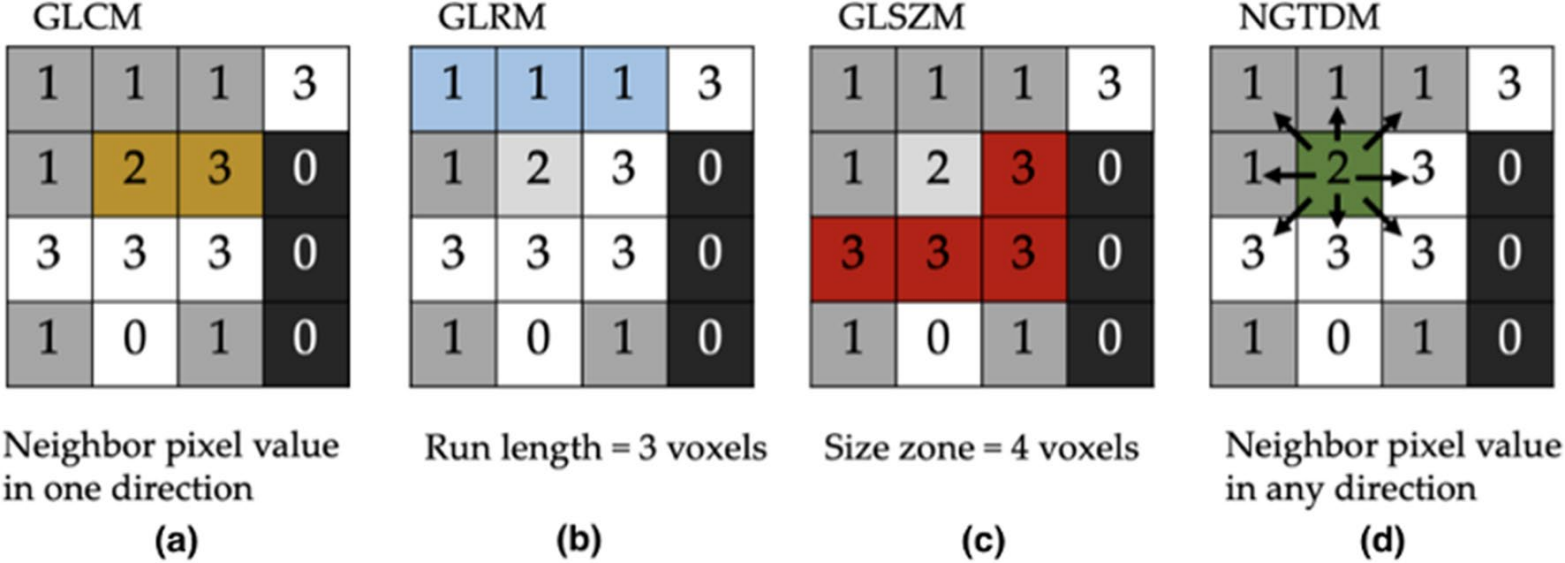
Examples of specific matrices allocating the information on the spatial distribution of pixel values in the image: (**a**) Gray Level Co-occurrence Matrix (GLCM), (**b**) Gray Level Run Length Matrix (GLRLM), (**c**) Gray Level Size Zone Matrix (GLSZM), (**d**) Neighboring Gray Tone Difference Matrix (NGTDM)

Most commonly used matrices are Gray Level Co-occurrence Matrix (GLCM), Gray Level Run Length Matrix (GLRLM), Gray Level Size Zone Matrix (GLSZM), Neighboring Gray Tone Difference Matrix (NGTDM), Gray Level Dependence Matrix (GLDM) and the Local Binary Pattern (LBP) (Fig. 2).

The GLCM contains statistical information about how pixel pairs are distributed in the image. The GLRLM considers higher-order statistical information and expresses the length of consecutive voxels having the same intensity in a pre-set direction in the image. The GLSZM quantifies gray level zones in an image, which are defined as the number of connected voxels that share the same gray level intensity. The region of interest is homogeneous when the matrix is wide and flat, and it is heterogeneous when the matrix is narrow. The NGTDM [6, 27] quantifies the difference between a gray value and the average gray value of its neighbors within a certain distance. The GLDM [28] quantifies gray level dependencies in an image, which are defined as the number of connected voxels within a certain distance that are dependent on the center voxel. LBP is a texture descriptor, introduced by Ojala [29], which assigns a label, i.e., a binary number, to each pixel in an image by comparing its gray level with the surrounding pixels. After labeling, an LBP histogram is obtained, with each bin representing one feature. As an example of application of Haralick features, it is worth mentioning a study performed to evaluate which Haralick's features are the most feasible in predicting tumor response to neoadjuvant chemoradiotherapy in colorectal cancer [30].

This classification of second-order features is not exhaustive because of the wide range of existing techniques. Some examples of second-order statistics features evaluated on the described matrices are reported in Table 4.

**Table 4:** Examples of second-order statistics features evaluated on each matrix with their description [23]

Group of features	Type of feature	Measure
Gray Level Co-occurrence Matrix (GLCM)	Correlation	Linear dependency of gray level values to their respective voxels in the GLCM
	Difference Entropy	a measure of the randomness/variability in neighborhood intensity value differences
	Difference Average	A measure of the relationship between occurrences of pairs with similar intensity values and occurrences of pairs with differing intensity values
	Difference Variance	A measure of heterogeneity that places higher weights on differing intensity level pairs that deviate more from the mean
	Joint Energy	A measure of homogeneous patterns in the image
	Joint Entropy	A measure of the randomness/variability in neighborhood intensity values
	Inverse Difference Moment (IDM), Inverse Difference Moment Normalized (IDMN) and Inverse Difference (ID)	A measure of the local homogeneity of an image
	Autocorrelation	A measure of the magnitude of the fineness and coarseness of texture
	Joint Average	The mean gray level intensity of the *i* distribution
	Cluster Prominence	A measure of the skewness and asymmetry of the GLCM
	Cluster Shade	A measure of the skewness and uniformity of the GLCM
	Cluster Tendency	A measure of groupings of voxels with similar gray level values
	Contrast	A measure of the local intensity variation, favoring values away from the diagonal
	Informational Measure of Correlation (IMC) 1 and 2 and Maximal Correlation Coefficient (MCC)	A quantification of the complexity of the texture
	Maximum Probability	Occurrences of the most predominant pair of neighboring intensity values
	Sum Average	A measure of the relationship between occurrences of pairs with lower intensity values and occurrences of pairs with higher intensity values
	Sum Entropy	A sum of neighborhood intensity value differences
	Sum of Squares	A measure in the distribution of neighboring intensity level pairs about the mean intensity level in the GLCM
Gray Level Run Length Matrix (GLRLM)	Short Run Emphasis (SRE)	A measure of the distribution of short run lengths, with a greater value indicative of shorter run lengths and more fine textural textures
	Long Run Emphasis (LRE)	A measure of the distribution of long run lengths, with a greater value indicative of longer run lengths and more coarse structural textures
	Gray Level Non-Uniformity (GLN) and Gray Level Non-Uniformity Normalized (GLNN)	A measure of the similarity of gray level intensity values in the image
	Run Length Non-Uniformity (RLN) and Run Length Non-Uniformity Normalized (RLNN)	A measure of the similarity of run lengths throughout the image
	Run Percentage (RP)	A measure of the coarseness of the texture by taking the ratio of number of runs and number of voxels in the ROI
	Gray Level Variance (GLV)	The variance in gray level intensity for the runs
	Run Variance (RV)	The variance in runs for the run lengths
	Run Entropy (RE)	a measure of the uncertainty/randomness in the distribution of run lengths and gray levels
	Low and High Gray Level Run Emphasis (LGLRE and HGLRE)	A measure of the distribution of low and higher gray level values
	Short and Long Run Low Gray Level Emphasis (SRLGLE and LRLGLE)	A measure of the joint distribution of shorter and long run lengths with lower gray level values
	Short and Long Run High Gray Level Run Emphasis (SRHGLE and LRHGLE)	A measure of the joint distribution of shorter and long run lengths with higher gray level values
Gray Level Size Zone Matrix (GLSZM)	Small and Large Area Emphasis (SAE and LAE)	A measure of the distribution of small and large size zones
	Gray Level Non-Uniformity (GLN) and Gray Level Non-Uniformity Normalized (GLNN)	A measure of the variability of gray level intensity values in the image
	Size Zone Non-Uniformity (SZN) and Size Zone Non-Uniformity Normalized (SZNN)	A measure of the variability of size zone volumes in the image
	Zone Percentage (ZP)	A measure of the coarseness of the texture by taking the ratio of number of zones and number of voxels in the region of interest
	Gray Level Variance (GLV)	The variance in gray level intensities for the zones
	Zone Variance (ZV)	The variance in zone size volumes for the zones
	Zone Entropy (ZE)	A measure of the uncertainty/randomness in the distribution of zone sizes and gray levels
	Low and High Gray Level Zone Emphasis (LGLZE and HGLZE)	A measure of the distribution of lower and higher gray level size zones
	Small Area Low and High Gray Level Emphasis (SALGLE and SAHGLE)	A measure of the proportion in the image of the joint distribution of smaller size zones with lower and higher gray level values
	Large Area Low and High Gray Level Emphasis (LALGLE and LAHGLE)	A measure of the proportion in the image of the joint distribution of larger size zones with lower gray level values
Neighboring Gray Tone Difference Matrix (NGTDM)	Coarseness	An average difference between the center voxel and its neighborhood and an indication of the spatial rate of change
	Contrast	A measure of the spatial intensity change, also dependent on the overall gray level dynamic range
	Busyness	A measure of the change from a pixel to its neighbor
	Complexity	A measure of non-uniformity and rapid changes in gray levels
	Strength	A measure of the primitives in an image
Gray Level Dependence Matrix (GLDM)	Small and Large Dependence Emphasis (SDE and LDE)	A measure of the distribution of small and large dependencies
	Gray Level Non-Uniformity (GLN)	The similarity of gray level intensity values in the image
	Dependence Non-Uniformity (DN) and Dependence Non-Uniformity Normalized (DNN)	The similarity of dependence throughout the image
	Gray Level Variance	The variance in gray level in the image
	Dependence Variance	the variance in dependence size in the image
	Low and High Gray Level Emphasis (LGLE and HGLE)	The distribution of low and high gray level values
	Small and Large Dependence Low Gray Level Emphasis (SDLGLE and LDLGLE)	The joint distribution of small and large dependence with lower gray level values
	Small and Large Dependence High Gray Level Emphasis (SDHGLE and LDHGLE)	The joint distribution of small and large dependence with higher gray level values

Higher-order statistics features can be obtained after applying filters or mathematical transforms to the images, giving rise to a virtually endless number of features. A lot of different radiomics features are continuously introduced, and an exhaustive review is almost impossible. However, some of them deserve a mention, such as fractals and SUV metric for PET specific applications [31, 32].

Features described so far are named “traditional features”. They are hand-crafted by human image processing experts and defined with an exact mathematical form. This differentiation is used to distinguish them from the so-called deep features. Deep learning algorithms are able to design and select the features themselves within its layers, without any need for human intervention.

### Imaging biobanks to collect and validate radiomics

As we have seen so far, there are many parameters that may influence the radiomics analysis, either via a direct causal association or exerting a confounding effect on statistical associations. Each result obtained in a radiomics study should be validated on an external and independent dataset. Data sharing among different institutions has become essential to translate radiomics from bench to bedside [33].

With this aim, infrastructures named imaging biobanks, defined as “organized databases of medical image collections associated with imaging biomarkers” [34–36], have begun to spread. Several European commission-financed projects are aiming to create virtual research infrastructures devoted not only to the storage and sharing of medical data but also to the deployment of new radiomics models. Among these projects, it is worth citing the ones described in Table 5, whose common goal is to create imaging biobanks on high-performance computing platforms and train deep neural networks with imaging and non-imaging data to build patient models. The final goal of such projects is to build a decision support system to predict risk of oncologic diseases, prognosis and response to therapy [37].

**Table 5:** European commission-financed projects dealing with creating medical imaging repositories and virtual research infrastructures devoted to the storage and sharing of data and to the deployment of radiomics algorithms

Project	Description
PRIMAGE [37]	PRedictive In-silico Multiscale Analytics to support cancer personalized diaGnosis and prognosis, Empowered by imaging biomarkers) project, mainly focused on childhood cancer
CHAIMELEON [38]	Accelerating the lab to market transition of AI tools for cancer management
Procancer-I [39]	An AI Platform integrating imaging data and models, supporting precision care through prostate cancer’s continuum
EuCanImage [40]	A European Cancer Image Platform Linked to Biological and Health Data for Next-Generation Artificial Intelligence and Precision Medicine in Oncology
INCISIVE [41]	A multimodal AI-based toolbox and an interoperable health imaging repository for the empowerment of imaging analysis related to the diagnosis, prediction and follow-up of cancer
EuCanShare [42]	An EU-Canada joint infrastructure for next-generation multi-Study Heart research

### Features analysis

The extraction methods generate from dozens to thousands of features, producing a high-dimensional feature space. But the more features we have, the more complex the classification model becomes. Furthermore, many features can be redundant or irrelevant, hindering the classification performance of the algorithms and yielding issues of dimensionality. Reducing the number of features speeds up the testing of new data and makes the classification problem easier to understand, improving the performance.

Therefore, radiomics analysis includes the main step of feature selection. This step consists in the exclusion of non-reproducible, redundant and non-relevant features to choose the most relevant ones for a specific application. Multiple ways for dimension reduction and feature selection exist, based both on conventional statistical methods and machine learning.

Some methods that are worth mentioning are Filters methods, Wrapper methods, Embedded methods and Unsupervised approaches [43–45]. Filters do not test any particular algorithm; they take into account the original features and select the top of them. They are especially based on correlation and mutual information criteria. Wrapper methods test a classification algorithm and search the subset of features that provides the best classification performance. Embedded methods are based on Machine Learning techniques that involve feature selection during the training stage. Some Unsupervised approaches are Cluster analysis, Principal component analysis (PCA), Isometric mapping (Isomap), locally linear embedding (LLE), diffusion map and t-Distributed Stochastic Neighbor Embedding (t-SNE).

Citing Guyon 2003 [45], “*The objective of variable selection is three-fold: improving the prediction performance of the predictors, providing faster and more cost-effective predictors and providing a better understanding of the underlying process that generated the data*.”

As this plethora of methods clearly shows, there is no universal “best” method for all tasks.

### Classifier model

Radiomics is a piece in the puzzle of precision medicine, its final goal being to build models able to classify the disease and/or to predict its outcome or the answer to a therapy. Thanks to a set of features, radiomics discovers patterns in large datasets using artificial intelligence, machine learning or statistical approaches. The limit between these different approaches to perform the classification task is blurred, and a precise categorization is virtually impossible, with mixed methods continuously arising. However, a distinction can be made between supervised and unsupervised methods [24, 46]. Supervised classifiers are trained using known information on the underlying pathology, learning to classify new patients with an unknown pathology [47–51]. Unsupervised methods do not use any pre-existing information, but they try to group the patients based on some form of distance metric, which is application-specific [47, 52–54]. It is worth mentioning the super learner [55], which is an ensemble machine learning algorithm that combines all of the models and model configurations that you might investigate for a predictive modeling problem and uses them to make a prediction as-good-as or better than any single model that you may have investigated.

Covariates used to train/validate/test the models can be genomic, proteomic, metabolomic profiles, histology, serum markers, patient histories and all the biomarkers related to the specific-use case.

Different metrics can be used to quantify the performance of the algorithm, depending on its class, such as accuracy, sensitivity, specificity, recall or silhouette and Davies-Bouldin index [56] for clustering algorithms. Area Under the receiver operating characteristic Curve (AUC) or Concordance Index (CI) is very important performance indexes too.

### Deep learning models

Nowadays, deep learning is probably the most powerful tool for image analysis [57]. There is a growing interest in the so-called Deep Radiomics, which is basically radiomics based on deep learning algorithms, which do not require the intermediate feature extraction step as in classic radiomics. A deep neural network is able to directly extract the features from the image. Since the algorithm “looks” directly at the images, without intermediate operations related to feature calculations, no information loss or extra errors are introduced, and the overall process is less time-consuming. A wide variety of deep architectures can be used, and the three different steps of the radiomic workflow, i.e., feature extraction, selection and classification, can be performed by the same complex algorithm. The layered structure of deep neural networks can discover more complex patterns and more abstract features than a traditional machine learning algorithm does.

Convolutional Neural Networks (CNN) models are state-of-the-art in many medical classification problems [58]. For instance, in a previous study, the deep features extracted from a CNN model can visually distinguish benign and malignant lung tumors [59]. Other examples are the application of a CNN model with the goal of lung cancer survival prediction [60], to extract deep features from breast mammographic images [61], or the inception CNN used for detecting diabetic retinopathy [62]. Multiple CNN is also a particular architecture used in radiomics, which was explored, for example, for Alzheimer’s disease diagnosis using MRI [63]. Other models frequently applied in previous studies are the Recurrent Neural Networks (RNNs) to process sequential data and useful for monitoring the medical images obtained from follow-up examinations, and the long-short-term-memory (LSTM) models, explored for prostate cancer benign and malignant classification [64]. Also, the so-called generative models have been used in radiomics. Their objective is to learn abstract features from the data distribution to generate new samples from the same distribution, and their main task is tumor classification.

Some issues still remain due to the so-called black-box problem, which is a sort of lack of interpretability of the internal processes of the algorithms because of their deep multi-layer structure [65]. Many efforts are being made in the field of “explainability”, which is the extent to which the internal mechanics of a deep learning algorithm can be explained in human terms. Furthermore, having to learn the intrinsic representation directly from data, these kinds of algorithms need to be trained with a larger number of images and use more computational resources. Another current development is the mixing between traditional and deep learning radiomics [66]. These techniques exploit both the advantages of deep learning and the interpretability offered by hand-crafted approaches.

## Radiomics in clinical practice

Radiomics can be applied to any medical study where the use of an imaging technique is required. A meta-analysis carried out by Park et al., which analyzed the scientific quality of publications, with the radiomics quality score (RQS), and the methodology of data collection in radiomics studies, with the Transparent Reporting of a multivariable prediction model for Individual Prognosis Or Diagnosis (TRIPOD), highlighted that 91% of radiomics studies concern oncological applications, and that for the most part (81%) radiomics is studied for diagnostic purposes. In oncological studies, the main applications are the differential diagnosis between neoplasms, correlation with molecular biology and genomics, the prediction of survival and the evaluation of the response to treatment [67].

The high prevalence of radiomics studies in the field of oncologyic imaging is due to the availability of a great amount of imaging and non-imaging data, large clinical trials, and also by social and economic factors that push research in oncology [42, 68].

Besides oncology, another field of application is that of neuroimaging. Radiomics features obtained from brain MRI have shown a great potential to uncover disease characteristics in neurodegenerative disorders [69] or mental illnesses [70]. This field of application would require a separate review as there exists huge literature dealing with this topic. There are also relevant studies on radiomics analysis applied to cardiac imaging for characterization of cardiovascular diseases [71]. Quantitative analysis is also expected to increase the value of musculoskeletal (MSK) imaging. Computational analysis of radiomics and machine learning could be used to build diagnostic, prognostic or predictive models also in this field of application [15].

As for what concerns oncology, a brief overview of the latest achievements and radiomics studies on brain, prostate, breast and lung cancers is reported in the following. Radiomics is being exploited for patients with brain tumors, and a variety of studies have been especially performed on brain metastasis [72, 73]. At the current status, clinical application of automated image analysis based on PET/MRI radiomics are showing a great potential in the differentiation of treatment-related changes from brain metastases recurrence after radiotherapy, in the prediction of brain metastases origin, in the differentiation of brain metastases from glioblastoma and in treatment response assessment.

In prostate cancer management, the implementation of MRI radiomics approaches sounds promising [74] in detection or aggressiveness prediction of prostate cancer [75]. Regarding breast cancer, recent studies demonstrate that, by adding radiomics to the standard radiological workflow in the field of breast imaging, it would be feasible to improve diagnostic accuracy of well consolidated techniques such as mammography, tomosynthesis and MRI [76]. Radiomics-based approaches are used for a comprehensive characterization of the tumor, providing a potential tool to develop a model for breast cancer classification and prediction. Ultrasound techniques have also been exploited for radiomics analysis in predicting breast cancer [77]. Radiomics is also expected to increasingly affect the clinical practice of treatment of lung tumors [78, 79]. A myriad of new radiomics-based evidence for lung cancer has been published [80, 81]. In fact, models based on radiomics features from CT and PET have been applied successfully in a variety of applications, such as distinguish malignant from benign lesions, detection of nodules by combining Machine Learning with the extraction of radiomics features, prediction of histology and tumor stage, prediction of mutation at a genetic level and quantification of severity in diffuse lung disease. It is also relevant to mention the use of Artificial Intelligence and radiomics in sarcopenia evaluation [82]. In particular, a recent study revealed that chest CT radiomics combined with machine learning classifiers allows to identify sarcopenia in advanced non-small cell lung cancer patients, by using skeletal muscle radiomics as a potential biomarker for sarcopenia identity [83]. The last application field that is worth mentioning is the gastrointestinal application. A plethora of studies on the new advances of radiomics applied to CT and MRI for the evaluation of gastrointestinal stromal tumors have been published, and the consequent potential clinical applications have been discussed [84]. Among these studies regarding the gastrointestinal stromal tumors, it is worth citing an interesting strategy developed for pattern classification based on the integration of radiomics and deep convolutional features [85].

All the promising results obtained in these fields of application reveal the potential of radiomics, and the key role that this process of analysis could have in clinical practice. In particular, this tool may improve the accuracy of diagnoses and therapy response assessments with the advantage of avoiding invasive medical procedures in most cases. In this direction, radiomics could change cancer patient approach, by providing to the physician a non-invasive tool for diagnosis and prediction, based on the exploration of imaging biomarkers [86]. In addition, radiomics allows to derive patient-specific therapy and prognosis, fostering a personalized medicine approach.

### Challenges and potential solutions

As already mentioned, different limitations currently prevent an actual implementation of these radiomics-based techniques into clinical practice, hampering the effective support of clinical decision-making and the fostering of precision medicine. Some of them are the issues related to reproducibility and repeatability of radiomics features, data sharing and lack of standardization and proper validation and represent a real challenge for further research [87, 88].

Retrospective data suffer from non-harmonization problems because the images are often acquired with equipment from different vendors and therefore with different acquisition parameters, especially in multicentre studies [89, 90]. Different manufacturers use different acquisition, reconstruction techniques that can introduce differences between images and consequently features that are due only to technical differences, as radiomics results are highly sensitive to the processing parameters [91].

Data sharing combined with standardization of acquisition and reconstruction protocols could be a possible solution to this problem and may help in finding more robust radiomics features that can be validated on external datasets [92]. In this context, the new emerging field of imaging biobanks, defined as platforms enabling the access to imaging and related data, aggregated following a standard, looks promising. Since every single step of radiomics workflow affects the results and their reproducibility, the image biomarker standardization initiative (IBSI) has been proposed to work toward standardizing the extraction of image biomarkers from acquired imaging [21].

To introduce radiomics tools in clinical trials, there is the need to provide reliable results. Thus, it is fundamental to establish objective and common measures to evaluate the results and validate the performance of a radiomics study, ensuring its reliability.

Moreover, radiomics models based on deep learning are seen by the clinicians as black boxes, able to give good prediction outcomes for particular clinical applications but without providing an intelligible explanation [93]. Therefore, interpretability and explainability of these models are ongoing areas of research, with various tools being investigated [94].

### Radiogenomics

A more comprehensive interpretation of the imaging biomarkers could be certainly possible through their combination with other kinds of data. An imaging biomarker should be an objective indicator of normal biological processes, pathological processes or biological responses to a therapeutic intervention. Imaging phenotypes reflect the underlying genomics. Tissue imaging can correlate with other kinds of complementary information, such as the one from clinical reports, treatment responses and genomic/proteomic assays, and this correlation may reflect the global outlook of cancer [1].

This evolving branch of radiomics linking imaging features to gene expression is today known as radiogenomics [95, 96]. By combining quantitative imaging features with clinical, genomic and other information in a multi-omics study, it is possible to mine these data to detect and validate radiomics biomarkers and better understand their function and biological significance. In this direction, the role of biobanking enabling the access to multiple types of data is crucial. A recent challenge is that of reliably connecting the available imaging biobanks to tissue biobanks to create this integration of different data to provide a radiogenomics approach to the patient.

However, radiogenomics in imaging, used to identify the genomics of a disease through imaging biomarkers, without the need for a biopsy, has to be distinguished from Radiogenomics in radiotherapy, which refers to the study of gene mutations associated with radiotherapy response [95].

Despite several challenges, both technical and clinical, which still need to be addressed in this field, accurate radiogenomics models are already being presented, and they can provide insight into the tumor in a non-invasive manner [97].

## Conclusion

In the present study, a thorough review of the typical radiomics analysis process is offered. A detailed explanation of all the steps used in the extraction of quantitative data from medical images, and their subsequent analysis is proposed. The aim was to summarize the several methods currently used in the various steps of the workflow, providing a deep technical overview of the analysis conducted in each step. This description stresses how all the different processes that can be used deeply affect the results and how this could be a problem for the repeatability and reliability of the analysis. Furthermore, we highlighted the reasons why radiomics analysis is of unique importance, encouraging the scientific community in establishing benchmarks and fostering the effective use of these promising research tools also in clinical settings.

A brief overview of the latest and most relevant clinical applications of radiomics in oncology is presented, sorting through the possibilities of advancement in prediction, diagnosis and treatment evaluation mainly in the studies of brain, prostate, breast and lung cancers. Although it is almost impossible to explore all the different applications in a single review, our aim was to provide the reader a general idea of the extent of different possible application domains, which could make radiomics such a powerful quantitative analysis tool. The variety of the radiomics studies carried out in research show another key point, i.e., how radiomics could offer the physician a non-invasive tool for a personalized medicine approach to the patient, in particular with the development of radiogenomics. An important stressed issue is that of limitations and challenges related to reproducibility, data sharing and lack of standardization, for which potential solutions that would help, such as standardization strategies and data sharing development, have been addressed. Therefore, the take home message is that an enormous effort should be encouraged to overcome these limitations and move the field of radiomics toward clinical implementation, by using it as an effective support in clinical decisions.

To summarize, a deep look into radiomics has been proposed, from the detailed description of current methods and different types of features that can be analyzed, to a wide and overall view of applications and future research directions, with a particular emphasis on the evolving branches of imaging biobanking and radiogenomics. Only an in-depth and comprehensive description of current methods and applications can reveal the potential power of radiomics and the need to translate the successful outcomes in research into an effective tool suitable in clinical practice.
